# *In silico* clinical trials for anti-aging therapies

**DOI:** 10.18632/aging.102180

**Published:** 2019-08-24

**Authors:** Javier A. Menendez, Elisabet Cuyàs, Núria Folguera-Blasco, Sara Verdura, Begoña Martin-Castillo, Jorge Joven, Tomás Alarcón

**Affiliations:** 1 ProCURE (Program Against Cancer Therapeutic Resistance), Metabolism and Cancer Group, Catalan Institute of Oncology, Girona, Spain; 2 Girona Biomedical Research Institute (IDIBGI), Girona, Spain; 3 Quantitative Cell Biology Lab, The Francis Crick Institute, London, United Kingdom; 4 Unit of Clinical Research, Catalan Institute of Oncology, Girona, Spain; 5 Unitat de Recerca Biomèdica (URB-CRB), Hospital Universitari de Sant Joan, Institut d'Investigació Sanitària Pere Virgili, Reus, Spain; 6 ICREA, Barcelona, Spain; 7 Centre de Recerca Matemàtica (CRM), Barcelona, Spain; 8 Departament de Matemàtiques, Universitat Autònoma de Barcelona, Barcelona, Spain; 9 Barcelona Graduate School of Mathematics (BGSMath), Barcelona, Spain

**Keywords:** aging, cancer, inflammation, senolytics, biomathematics

## Abstract

Therapeutic strategies targeting the hallmarks of aging can be broadly grouped into four categories, namely systemic (blood) factors, metabolic manipulation (diet regimens and dietary restriction mimetics), suppression of cellular senescence (senolytics), and cellular reprogramming, which likely have common characteristics and mechanisms of action. In evaluating the potential synergism of combining such strategies, however, we should consider the possibility of constraining trade-off phenotypes such as impairment in wound healing and immune response, tissue dysfunction and tumorigenesis. Moreover, we are rapidly learning that the benefit/risk ratio of aging-targeted interventions largely depends on intra- and inter-individual variations of susceptibility to the healthspan-, resilience-, and/or lifespan-promoting effects of the interventions. Here, we exemplify how computationally-generated proxies of the efficacy of a given lifespan/healthspan-promoting approach can predict the impact of baseline epigenetic heterogeneity on the positive outcomes of ketogenic diet and mTOR inhibition as single or combined anti-aging strategies. We therefore propose that stochastic biomathematical modeling and computational simulation platforms should be developed as *in silico* strategies to accelerate the performance of clinical trials targeting human aging, and to provide personalized approaches and robust biomarkers of healthy aging at the individual-to-population levels.

A growing aging population burdened by multiple chronic diseases and geriatric syndromes has escalated to become a major public health and medical challenge in Western society [1]. Elderly individuals who are frail are less able to cope with physiological stressors, and not only experience suboptimal health outcomes but also contribute to rising healthcare costs [2,3]. Thus, the combined effects of increased life expectancy and the projected exponential growth of the aging population will critically reduce the capacity of our medical care systems to improve healthcare quality in the elderly. In this scenario, the traditional biomedical focus on single diseases, which often reduces mortality without preventing or reversing the decline in overall health, will result in an inadequate resolution of the societal burden of chronic illnesses and frailty [1–4].

A better understanding of how a limited number of fundamental processes drives aging has led to the emergence of candidate drugs and non-pharmacological strategies specifically targeting the so-called hallmarks of aging [5–9]. It is safe to predict that the next 10 to 20 years will see a range of aging-related treatments clinically tested for their ability to lower all-cause mortality by targeting complex multimorbidity (i.e., *healthspan promoters*), to boost the capacity of cells and tissues to respond or recover from an acute health stress (i.e., *resilience promoters*), or even to reverse functional decline *via* organismal rejuvenation (i.e., *lifespan promoters*). Although some of these drugs are already in clinical use for other specific indications (e.g., metformin and rapamycin) [10,11], the design of *bona fide* anti-aging clinical trials with the existing (and with a yet-to-be funded) pipeline of healthspan-/resilience-/lifespan-promoting treatments will pose a unique set of challenges, namely: a.) the occurrence of intra- and inter-individual variability of response to aging-targeting strategies with a consequent need for personalized treatments; and b.) the need for reasonable end points and robust biomarkers of healthy aging to assess the response to aging-targeted interventions at the individual-to-population levels.

## The *trade-offs* of aging-targeted strategies: the epigenetic nature of a convergent concern

The majority of existing therapeutic strategies targeting aging can be broadly grouped into four categories: systemic (blood) factors (including parabiosis), metabolic manipulation (diet regimens [DR] and dietary restriction mimetics [DRMs]), suppression of cellular senescence (senolytic drugs), and cellular (partial/transient) reprogramming (reviewed in [8]). A comparison of their underlying mechanisms of action (e.g., inflammation, nutrient-sensing pathways, epigenomic remodeling, autophagy, mitochondria) and target cells (e.g., stem cells, connective tissue and vasculature cells, senescent cells) immediately suggests their potential combinatorial value. Accordingly, it is tempting to suggest that shared mechanisms or target cells could be exploited to induce more direct anti-aging effects, while different mechanisms could be targeted in combination to enhance the effects.

**Figure 1:**
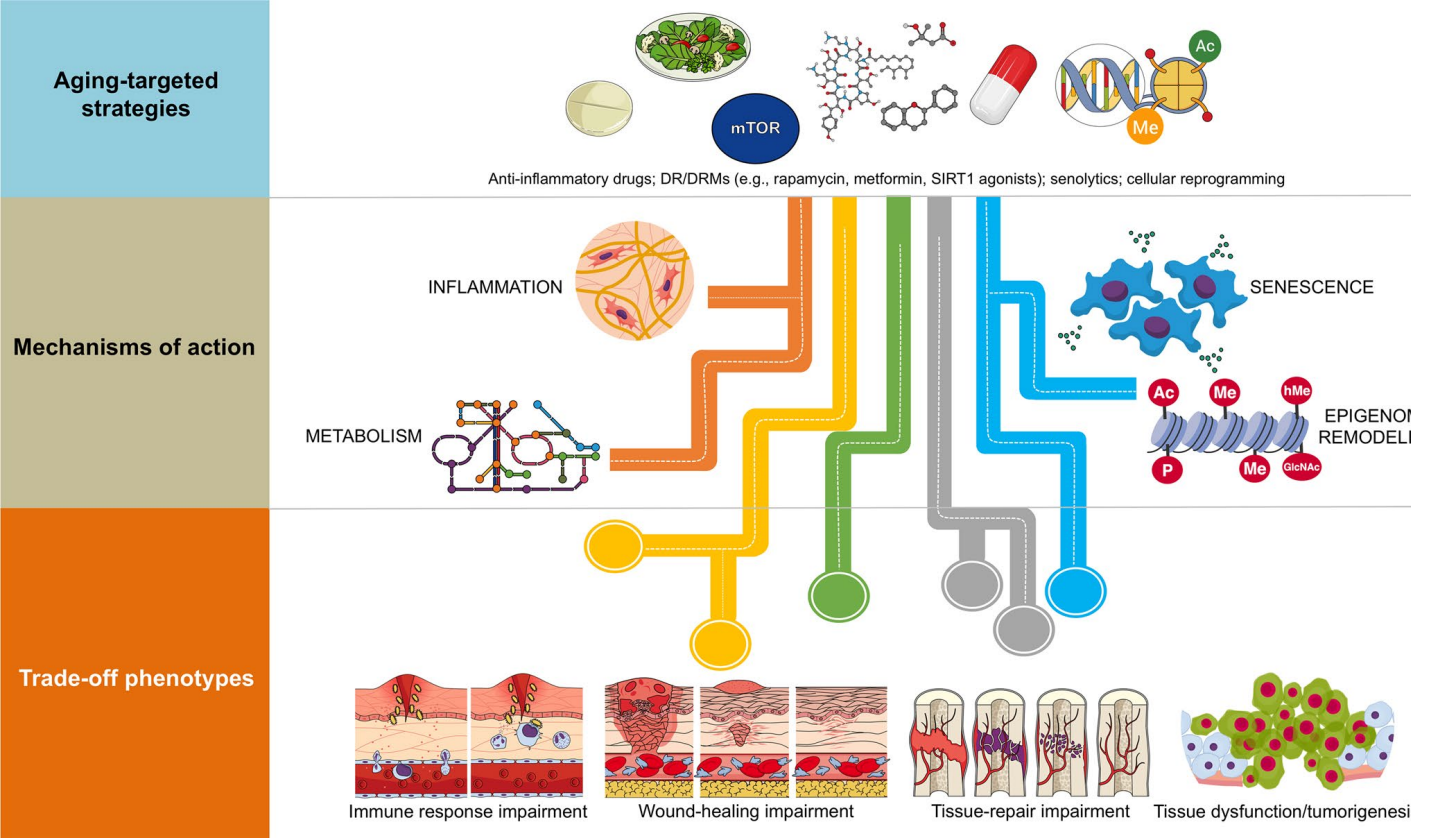
**Combinatorial anti-aging strategies: Good and bad directions.** (**A**) Chronic inflammation (or inflamm-aging) is commonly viewed as a central feature in aging and aging-related diseases [6,7,43]. Accordingly, the benefits arising from the anti-inflammatory effects of either dietary interventions/DRM strategies or senolytics might be directly enhanced in combination with well-characterized anti-inflammatory drugs (e.g., aspirin, non-steroidal anti-inflammatory drugs [NSAIDs] or nordihydroguaiaretic acid) [44–49], which may even operate at the hypothalamus level to impede systemic inflammation-driven aging [50]. However, a new paradigm for the role of inflamm-aging and immunosenescence (an aging-related decline in immune parameters that often leads to subclinical accumulation of pro-inflammatory factors and inflamm-aging) in the aging process begins to suggest that successful aging and longevity can only occur when changes in inflamm-aging are balanced by compensatory anti-inflamm-aging mechanisms [51]. In such a scenario, in which the immune/inflammatory system is adapted/remodeled to provide the best possible anti-pathogen protection when the adaptive immune systems fails in the elderly/aged organisms, the preservation of such apparently detrimental changes may be needed for optimal healthspan/longevity. Therefore, the aforementioned combinations (generating exacerbated decreases of the two components of the immunosenescence/inflamm-aging duo) might cause potential harm in terms of immune response impairment to infections in aged individuals. (**B**) Because many of the aging-associated features that are reverted by partial cell reprogramming are related to senescence [25], and given that cellular reprogramming on its own has been shown to rejuvenate senescent cells [52], a combination of senolytic and reprogramming strategies might provide synergistic anti-aging effects. However, it should be noted that the presence of inflammatory factors such as interleukin-6 (IL-6) in response to injury-induced senescence promotes cellular plasticity and responsiveness of neighboring cells to *in vivo* reprogramming-like phenomena [23,24]. Indeed, specific pharmacological and genetic removal of senescent cells has been shown to impair *in vivo* reprogramming efficiency [53]. The routes, kinetics, and intensities that would distinguish between the beneficial and the harmful effects of senescence in terms of the inherent susceptibility of different cell and tissue types to cellular reprogramming remains an important direction for future studies. (**C**) The cellular epigenome landscape directs cell fate and reflects its health and biological age. Certain dietary interventions (e.g., ketogenic diets), by altering the availability of key regulators for chromatin-modifying enzymes (e.g., the histone deacetylases inhibitor β-hydroxybutyrate), may provide a direct link between metabolism and epigenomic remodeling to extend healthspan and longevity [54,55]. Given the ability of dietary interventions and DRMs to affect the epigenome [31,56–58] and to suppress the development of senescence [59–62], an intriguing possibility is that dietary, pharmacological, and behavioral strategies targeting nutrient-sensing pathways might synergistically interact with cellular reprogramming strategies to provide better anti-aging outcomes. Nonetheless, certain dietary interventions could promote enhanced stemness and tumorigenicity in specific stem cell compartments and differentiated cell types [63,64]. Interference with normal wound healing processes and potentiation of tumorigenesis and cancer progression might therefore occur after excessive perturbation of the epigenome plasticity, as a maladaptive response to a combination of metabolic manipulations and partial reprogramming.

The attractive possibility of achieving supra-additive outcomes when therapeutically addressing human aging with combinatorial strategies should consider the parallel existence of constraining *trade-off* phenotypes (Figure 1). For example, because senescent cells are known to have beneficial effects including facilitating tissue repair after injury and preventing tissue fibrosis, an unrestricted inhibition of senescence and/or inflammation with the use of senolytics could significantly impair the ability of the organism to undergo normal tissue repair and remodeling while promoting tissue-specific fibrosis [12–16]. Also, DR/DRM strategies can suppress senescence and pro-inflammatory cytokine levels, potentially interfering with normal wound healing processes [17,18]. Chronic suppression of senescence, a primary mechanism of tumor suppression [19,20], and unrestricted cellular reprogramming, might similarly lead to excessive distortion of the epigenome landscape, loss of cell identity and tumorigenesis [21,22]. It therefore appears that the disruption of entry-exit mechanisms and kinetics of endogenous injury-repair mechanisms is the convergent *trade-off* of all the emerging anti-aging strategies. Accordingly, whereas transient epigenomic remodeling in response to partial cellular reprogramming phenomena is a rejuvenation strategy for erasing the hallmarks of aging at the molecular and cellular level [23–26], tissue repair impairment and tumorigenesis could also occur as the trade-off if the unrestricted epigenetic remodeling in response to aging-targeted strategies is not accompanied by self-repair of injury or disease [27].

## Endogenous heterogeneity and individual-to-individual variability: two key regulators of the benefit/risk ratio of anti-aging strategies

Beyond genetic factors, non-genetic stimuli such as inflammation and metabolic disturbances can promote an overly restrictive epigenetic landscape capable of blocking normal differentiation processes or preventing the induction of tumor suppression programs. Conversely, these cues can induce overly plastic epigenetic states capable of stochastically promoting inadequate cell fate transitions or activating oncogenic programs [28–31]. This may allow for tissue-specific responses to changing environmental conditions and, perhaps more importantly, it implies that systemic signals can be interpreted by local injury-repair mechanisms. For example, whereas defective insulin signaling at the systemic level associates with aging, tissue dysfunction, and impaired wound healing, a local potentiation of insulin sensitivity in the liver suffices to switch the hepatic injury response from a stromal repair process to a more beneficial epithelial repair process [32]. The ability of the microenvironment, including the stem cell niche, to locally integrate systemic signals and alter the type and intensity of repair mechanisms strongly suggests that anti-aging strategies should restore a homeostatic balance of sub-cellular transducers (e.g., epigenome, mitochondria), cellular functions (e.g., stem cells, senescent cells, vascular and connective tissue cells –the latter present throughout the entire organism), and systemic features (e.g., inflammation, nutrient sensing), in order to dynamically maintain organismal function.

**Figure 2:**
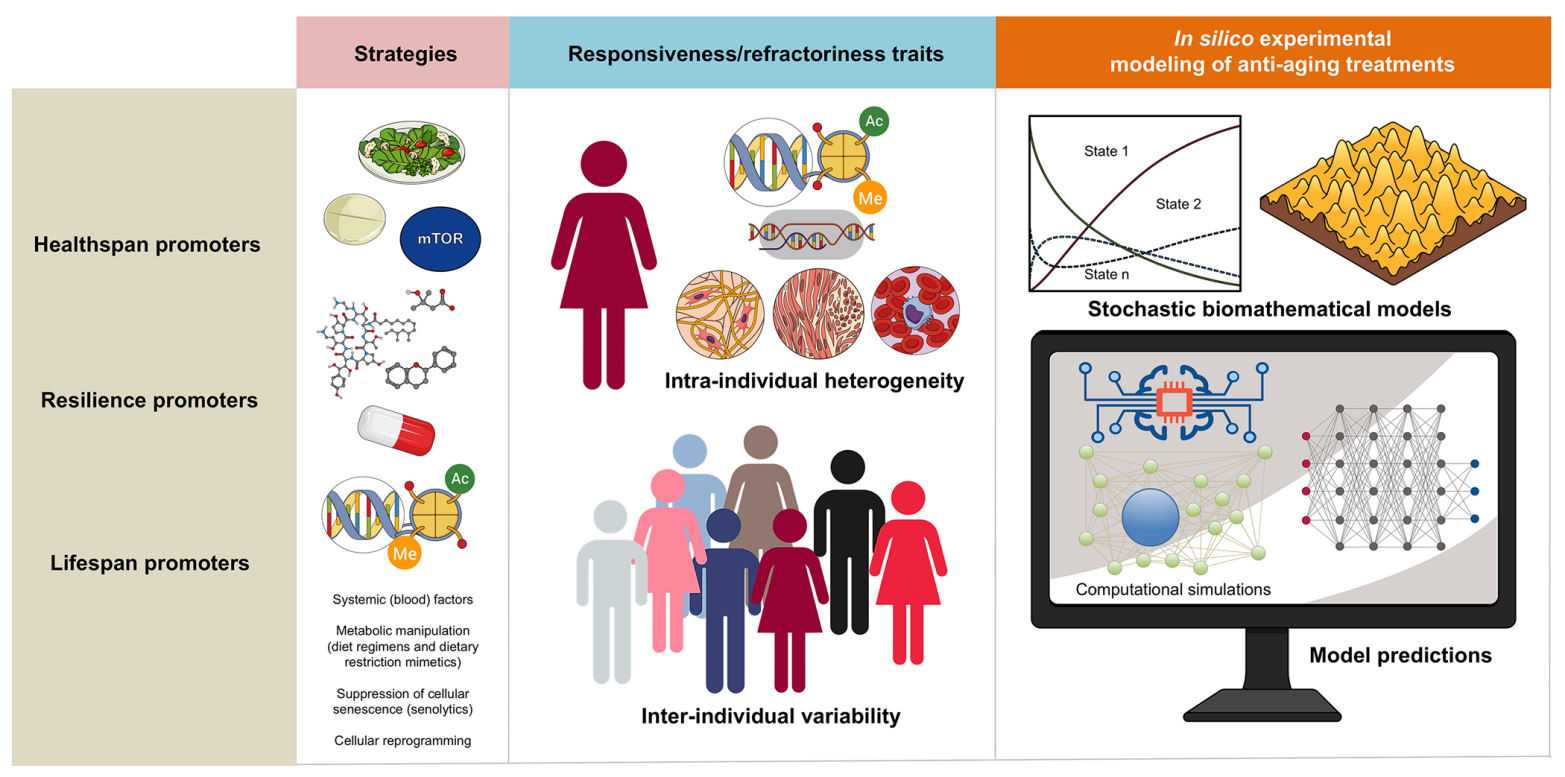
**Biomathematical approaches to accelerate the performance and personalize the use of aging-targeted strategies.** The benefit/risk ratio of aging-targeted interventions largely depends on intra- and inter-individual variations of susceptibility to the healthspan-, resilience-, and/or lifespan-promoting effects of the interventions. Stochastic biomathematical modeling and computational simulation platforms should be developed as *in silico* strategies to accelerate the performance of clinical trials targeting human aging, and to provide personalized approaches and robust biomarkers of healthy aging at the individual-to-population levels.

Endogenous heterogeneity and individual variability might operate as the fundamental epigenetic dimensions determining the tissue and organismal potential to benefit from anti-aging strategies (Figure 2). The benefit/risk ratio of anti-aging strategies targeting inflammation (e.g., senolytics) or nutrient/metabolism-sensing pathways (e.g., DR/DRMs modulating the communication between metabolism and epigenetics) might be dictated by the pre-existing degree of epigenetic plasticity and phenotypic malleability of the different cell populations in aging tissues. Accordingly, the occurrence of intra- and inter-individual variability driven by sub-cellular/cellular (e.g., local availability of epigenetic substrates/cofactors and/or metabolic cues) and more systemic features (e.g., inflammation driven by senescent cells, activated fibroblasts or endothelial cells) begins to be understood as a consequence of different aging trajectories occurring in different tissue cell subpopulations [33]. This likely reflects changes in the short-range/paracrine communication between distinct cell types within a tissue, as well as between different individuals. Unfortunately, we are lacking robust and standardized approaches to capture such fundamental stochastic aspects of aging biology.

## Biomathematics to capture the stochasticity of aging biology

**Figure 3:**
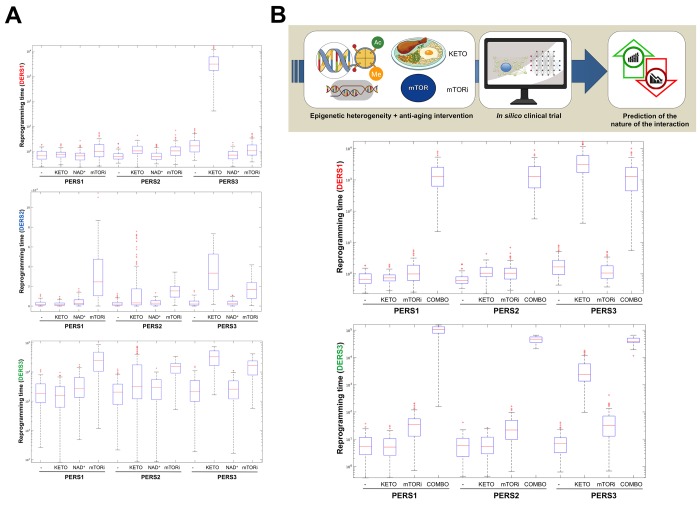
**Baseline epigenetic heterogeneity and efficacy of anti-aging strategies: A proof-of-concept *in silico* trial.** We have recently presented a stochastic biomathematical modeling and computational simulation strategy that might be incorporated as a valuable tool for assessing the benefit/risk ratio of therapeutic approaches aimed to target the aging/cancer-related perturbations of the epigenome [35]. Briefly, we quantified the heterogeneity and robustness of differentiation epigenetic and pluripotency regulatory systems (DERS and PERS, respectively) in terms of the average reprogramming time associated with differentiation-primed (benefit) and pluripotency-locked (risk) states. Such calculation can therefore be employed as a proxy of the expected efficacy of a given anti-aging strategy (i.e., longer reprogramming times associate with more efficient anti-aging outcomes). Regarding DERS heterogeneity, we observed three different clusters associated with a differentiated-primed behavior (DERS1), a differentiation-refractory (stem-like) behavior (DERS2), and an indecision behavior (DERS3). Several kinetic parameters associated with histone deacetylase (HDAC, c_11_) and histone acetylase (HAC, c_15_ and c_16_) activities sufficed to account for the distinction between DERS clusters [35]. Regarding PERS heterogeneity, we observed three different clusters defined by the large (PERS1), intermediate (PERS2), and small (PERS3) values of the average waiting time needed for transitioning from closed to open ER states [35]. As a proof of concept, here we considered nine combinatorial scenarios corresponding to all the possible combinations between three different differentiation DERS and PERS challenged with three different anti-aging strategies (**A**), namely ketogenic diet (mimicked by reducing by 50% the kinetic parameter associated with HDAC activity [i.e., c_11_]), NAD^+^ boosters (mimicked by increasing by 50% the kinetic parameter associated with HDAC activity [i.e., c_11_]), and mTOR inhibition (mimicked by reducing by 25% the two parameters associated with HAC activity [i.e., c_15_ and c_16_]). Whereas ketogenic diet- and mTOR inhibition-like strategies were notably found to be more efficient than NAD^+^ boosting-like approaches across all the baseline epigenetic scenarios, it was noteworthy that the most sensitive one was that defined by the combination of PERS3 with DERS1 (**A**). Based on these results, we decided to assess the direction and intensity of the average reprogramming time when combining ketogenic diet- and mTOR inhibition-like strategies (**B**). With the exception of baseline scenarios where the strong single-agent positive outcome left little room for additional gains, we observed that strong, synergistic interactions tend to be more specific to particular epigenetic states than were single strategies (**B**). Our *in silico* approach exemplifies how baseline epigenetic heterogeneity might dictate not only the positive outcomes of single aging-targeted therapies such as ketogenic diet and mTOR inhibition but also the degree of positive synergistic effects that were predicted to occur upon concurrent targeting of an apparently existing HDAC-mTOR cross-link.

Biomathematical and computational systems biology are expected to deconstruct, model, and simulate the power that epigenetic landscapes have on the susceptibility of cells and tissues to retain or lose their normal identity and function. We recently presented a theoretical framework in which the acquisition of phenotypic plasticity (*via* transient disruption of the homeostatic resilience of the chromatin structure and functioning) followed by reparative differentiation phenomena (replacement or dilution of damaged/diseased cells) might constitute a common route through which aging-targeted therapies could enhance the organismal self-repair capacity after damage, stress, and disease [27]. This conceptualization of aging as a senescence-inflammation regulatory phenomenon of phenotypic plasticity and reparative reprogramming actually incorporates the concept of frailty, which can be viewed not only as a deficit accumulation, but also as a reduction in the molecular/cellular/physiological reserves of the aged organs/organism, leading to less efficient responses to stress. A predictive computational model of cell fate reprogramming revealed that pathological cell states, and their suppression, might result from the sole stochastic translation of metabolic cues (e.g., DR and DRMs) into resilient/plastic cell states [34]. A more advanced stochastic, multiscale reduction method of combined epigenetic regulation-gene regulatory network has mathematically captured how epigenetic heterogeneity can operate as the driving force governing the routes and kinetics to entry and exit from unrestrained epigenetic plastic states [35]. Moreover, we were able to computationally validate the likelihood of unlocking aberrant cell states disabled for reparative differentiation and prone to malignant transformation (and drive their function to their correct repair function) solely by manipulating the intensity and direction of a few epigenetic control switches [35]. Altogether, these findings provided support to the notion that predictive mathematical modeling and computational simulation platforms might be incorporated as new tools to optimize the clinical success of aging-targeting therapeutics while providing individualized ways to target human aging. By analyzing computationally-generated proxies of the impact of aging-targeted strategies on the behavior of epigenetic-regulatory (ER) systems controlling cell fate reprogramming, we here exemplify how baseline epigenetic heterogeneity might dictate not only the efficacy of single anti-aging therapies such as ketogenic diet (as an archetypal lifespan/healthspan-promoting dietary approach leading to inhibition of histone deacetylases [36–38]) and mTOR inhibition (as an archetypal lifespan/healthspan-promoting metabolic approach that couples nutrient sensing to epigenetic regulation by promoting a decrease in histone acetylation [39,40]) but also the degree of an *in silico* predicted anti-aging synergistic response to a ketogenic-like dietary regimen concurrent with mTOR blockade (Figure 3).

## Stochastic biomathematical platforms to strengthen the efficacy and personalization of healthspan/resilience/lifespan-promoting therapies

The aforementioned computational models are consistent with a scenario in which cell/tissue plasticity and resilience are fundamental epigenetic phenomena associated with phenotypic variation that drives successful repair, degeneration or transformation. Because such models predict that the fine-tuning of certain signals might re-direct plastic epi-states into phenotypic resilience or vulnerability, they seem to be idoneous to *in silico* test the ability of a given aging-targeted intervention to promote tissue repair impairment, tissue-specific fibrosis or dysfunction (including tumorigenesis) because of loss of cellular identity. Such stochastic biomathematical platforms would comprise a machine-learning/computational component to uncover the phenotypic behavior of cell populations mimicking the intra- and inter-individual heterogeneity and variability when challenged with aging-targeting strategies (Figure 2). Computational tools suited for complex stochastic dynamical systems could be employed to *in silico* construct and comparatively integrate the shape and evolution of epigenome remodeling upon such imposition of aging-targeted approaches. Quantitative probabilistic simulations could be then incorporated to functionally infer the proportions and interconversion rates of the existing communities of epi-states. Ideally, a multi-scale theoretical integration of epi-phenotype dynamics prospectively coupled to confirmatory laboratory-based testing of the *in vivo* amelioration of aging hallmarks, would allow an iterative improvement process capable not only of predicting the effectiveness but also of personalizing aging-targeting interventions. Indeed, by computationally determining the key regulatory nodes and kinetic routes of phenotypic plasticity in an unbiased and systematic manner, stochastic biomathematical platforms would provide end-points and biomarkers of healthy aging when assessing the individual-to-population levels of response to aging-targeted interventions.

## The unique features of clinical trials targeting human aging

The ultimate goal of aging-targeted strategies extends beyond an isolated impact in each separate age-related disease. Measuring the global impact of such healthspan-/resilience-/lifespan-promoting interventions on “hard” composite outcomes such as burden of chronic diseases, functional dependence, and/or mortality, will require a major effort in the design of controlled clinical trials in large, heterogeneous, elderly populations. Before committing to complex, long-term studies, a series of intermediate, smaller clinical trials of a given aging-targeted intervention should provide experimental evidence for the concept that basic aging-driving mechanisms can be therapeutically targeted. Moreover, because aging is not an indication currently recognized by the reference regulatory agencies (FDA, EMA), a key goal for forthcoming clinical trials will be to define outcomes representative of fundamental aging features that might be acceptable as potential indications. Such *in human* amelioration of aging hallmarks might include multi-morbidity, frailty and functional decline. In this regard, two broad strategies involving short-duration or pilot studies have been proposed to solidify the rationale and better inform the design and scale of larger and longer trials [2]. The so-called “extending healthspan” study design is based on the notion that, if aging is associated with a gradual accumulation of multiple chronic diseases, geriatric syndromes, and functional decline, those interventions directly targeting the aging machinery should have a significant effect on multi-morbidity. Conversely, the so-called “enhancing resilience” study design is based on the notion that, if the ability of response to or recovery from an acute health stress back to the functional/healthy baseline declines with age, those interventions directly targeting the aging machinery should be able to enhance the capacity of an individual to recover functional independence. The question remains as to whether rejuvenation interventions contributing to “lifespan extension” such as transient reprogramming, which has been observed exclusively in the context of premature aging [25,26], may also prove beneficial for whole-organism rejuvenation in the absence of injury or disease and/or in a naturally aged context.

Even in the context of broad strategies such as the proof-of-concept studies described above, the sole testing of existing drug pipelines (e.g., sirtuin agonists, senolytic agents, DRMs including metformin, mitochondrial function and autophagy regulators, anti-hypertensive agents, etc) and non-pharmacological strategies (e.g., blood factors, DRs, partial cellular reprograming, inducible telomerase, exercise, fecal microbiota transplantation, etc) might generate significant challenges for clinical trial design regarding population selection, study length and type of intervention, and measurement of the various outcomes for each candidate. Accordingly, a recent study has raised concerns about broad recommendations for the use of metformin as a potential healthspan extending treatment [10,41] in healthy individuals [42]. The influence of metformin on aerobic exercise training-induced improvements in physiological functions in older adults was highly variable, with the occurrence of positive and negative responders being associated with the heterogenous effect of metformin on the mitochondria [42]. These data strongly support the notion that prior to prescribing any aging-targeted intervention, additional studies are needed to understand the mechanisms that elicit positive and negative responses before assuming synergism of combining such strategies. In this scenario, we propose that the development of predictive mathematical models and computational simulation platforms to operatively integrate the multi-scale epi-phenotypes from single cells to individuals and populations might enhance the clinical performance of aging-targeting therapeutics by helping to circumvent the *trade-offs* of aging-targeted interventions while providing individualized ways to target human aging (Figure 3).

## Aging-targeted interventions: challenging an old paradigm

The conventional paradigm “*one disease*, *one drug*” should be updated to achieve the vision of targeting aging as a common component of human diseases. The current deterministic genetic paradigm of diagnosing and treating each separate age-related disease fails to fit with the broader anti-aging strategies aimed to address the closely related concepts of healthspan, resilience, and lifespan, which should be therapeutically managed in the absence of discrete, targetable genetic drivers of aging progression. Perhaps more importantly, current frameworks cannot capture the stochastic aspects that drive the shared trade-offs of the emerging strategies for organismal healthspan and rejuvenation, namely tissue-repair/wound-healing impairment and tumorigenesis.

Successful clinical trials with new families of candidate interventions targeting the biologic machinery of aging *per se* would be groundbreaking; delaying, preventing (or even reversing) the aging process would result in tremendous cost savings for healthcare systems while increasing the productive contributions that could be made by the older members of our societies. By modeling and predicting the behavior of interventions that target the aging hallmarks in both long-term and acute settings, defined by extension of healthspan/lifespan and enhanced resilience to acute stressors (i.e., reduced frailty), respectively, robust and standardized approaches such as stochastic biomathematical platforms would have the ability to sidestep most of the current challenges in aging-targeting clinical trials, to accelerate the achievement of optimum health and life quality in aging populations.
